# The effect of amyloid deposition on longitudinal resting-state functional connectivity in cognitively normal older adults

**DOI:** 10.1186/s13195-019-0573-1

**Published:** 2020-01-06

**Authors:** Chemin Lin, Maria Ly, Helmet T. Karim, Wenjing Wei, Beth E. Snitz, William E. Klunk, Howard J. Aizenstein

**Affiliations:** 1 Department of Psychiatry, Keelung Chang Chung Memorial Hospital, Keelung, Taiwan; 2 Department of Psychiatry, University of Pittsburgh, Pittsburgh, PA USA; 3 The Third Xiangya Hospital of Central South University, Changsha, Hunan China; 4 Shanghai Mental Health Center, Shanghai Jiao Tong University School of Medicine, Shanghai, China; 5 Department of Neurology, University of Pittsburgh, Pittsburgh, PA USA; 6 Department of Bioengineering, University of Pittsburgh, Pittsburgh, PA USA; 7 Western Psychiatric Institute and Clinic, 3811 O’Hara Street, Pittsburgh, PA 15213 USA

**Keywords:** Preclinical Alzheimer’s disease, Resting-state fMRI, Longitudinal, Compensation, Homeostatic regulation, Amyloid, PiB, Connectivity

## Abstract

**Background:**

Pathological processes contributing to Alzheimer’s disease begin decades prior to the onset of clinical symptoms. There is significant variation in cognitive changes in the presence of pathology, functional connectivity may be a marker of compensation to amyloid; however, this is not well understood.

**Methods:**

We recruited 64 cognitively normal older adults who underwent neuropsychological testing and biannual magnetic resonance imaging (MRI), amyloid imaging with Pittsburgh compound B (PiB)-PET, and glucose metabolism (FDG)-PET imaging for up to 6 years. Resting-state MRI was used to estimate connectivity of seven canonical neural networks using template-based rotation. Using voxel-wise paired *t*-tests, we identified neural networks that displayed significant changes in connectivity across time. We investigated associations among amyloid and longitudinal changes in connectivity and cognitive function by domains.

**Results:**

Left middle frontal gyrus connectivity within the memory encoding network increased over time, but the rate of change was lower with greater amyloid. This was no longer significant in an analysis where we limited the sample to only those with two time points. We found limited decline in cognitive domains overall. Greater functional connectivity was associated with better attention/processing speed and executive function (independent of time) in those with lower amyloid but was associated with worse function with greater amyloid.

**Conclusions:**

Increased functional connectivity serves to preserve cognitive function in normal aging and may fail in the presence of pathology consistent with compensatory models.

**Electronic supplementary material:**

The online version of this article (10.1186/s13195-019-0573-1) contains supplementary material, which is available to authorized users.

## Introduction

Alzheimer’s disease (AD) is an age-related neurodegenerative disease affecting approximately 5.5 million people and is the sixth leading cause of death in the USA. As the baby boomer population is rapidly aging, this number could rise to as high as 7 million by 2050 [1]. Considering this increase in prevalence, it is becoming increasingly more important to understand the neurobiological effects of AD that may inform treatment and prevention strategies.

The pathophysiological processes contributing to AD begin decades prior to the onset of clinical symptoms [2]. This period is referred to as preclinical AD where an individual is cognitively normal but demonstrates in vivo amyloid burden. It is important to note that preclinical AD does not necessarily imply ultimate progression to an AD dementia diagnosis. The prevailing model of AD progression hypothesizes that amyloid-beta (Aβ) deposition is the first detectable biomarker indicating an individual’s risk for developing AD, which occurs in this preclinical stage [3]. In this preclinical stage and prior to cognitive impairment, previous studies have shown that greater amyloid load is associated with differences in resting-state functional connectivity [4–8].

Among resting-state networks, the default mode network is affected to a greater extent in mild cognitive impairment (MCI) and AD [4]. During preclinical stages, greater amyloid is associated with low functional connectivity in the posterior default mode network (DMN) indicating this change may occur very early on [9]. Further, the DMN connectivity may be associated with early amyloid deposition, the topology of which largely overlaps with the DMN—possible evidence of amyloid toxicity. These changes have been interpreted to represent disruption of local networks, as well as “compensatory” reorganization [4–8] since cognitive function is largely intact in preclinical AD. However, these cross-sectional studies have not shown intra-individual change in functional connectivity over time or the effect of amyloid deposition on that process.

The association between cognitive function and both amyloid and connectivity is unclear. Cross-sectional studies have found that in the preclinical stage, functional connectivity was not correlated with concurrent cognitive function [10–12] and a meta-analysis demonstrate weak associations between amyloid and episodic memory in cognitive normal elderly participants [12]. However, we have previously demonstrated that in cognitively normal elderly individuals who were Pittsburgh compound B (PiB) positive, lower episodic memory was associated with greater default mode connectivity [9]. Further, baseline amyloid deposition predicts longitudinal cognitive decline [13, 14]. These mixed findings may be due to the state-dependent nature of this disease: we hypothesize that as amyloid accumulates in the earliest stages there is a natural compensatory response in connectivity that may help maintain cognitive function; however, this compensatory response is limited and may fail at some level of pathological burden that may lead to future cognitive decline.

We investigated the longitudinal effect of amyloid deposition on resting-state functional connectivity in cognitively normal older adults. We aimed to (1) identify changes in connectivity longitudinally, (2) investigate the role of amyloid in those changes, and (3) determine their associations with cognitive function.

## Methods

### Participants and study design

This project was a part of an ongoing study that aimed to understand amyloid pathology and subsequent cognitive decline in community-dwelling individuals with normal cognitive function [15]. Participants were recruited mostly through advertisements in the Pittsburgh Senior News, while other participants were recruited through the following: letters to participants who had completed previous studies, recruitment through another study (MyHat: NIA R01AG052521), word of mouth, and a website at the University of Pittsburgh (Pitt + Me). We included participants who were older than 65 years of age at baseline visit, fluent in English who had normal cognitive function (most neuropsychological test scores within 1 standard deviation [SD] after adjusting for age and education). We excluded participants with a diagnosis of mild cognitive impairment or dementia, history of major psychiatric or neurologic disorders, unstable medical conditions or medications that may affect cognitive function, sensory deficits that preclude cognitive testing, and contraindications to MRI. Participants underwent annual neuropsychological testing and biannual imaging studies with MRI, Pittsburgh compound B (PiB)-PET, and ^18^F-fluorodeoxyglucose (^18^F-FDG)-PET. This study was approved by the University of Pittsburgh Institutional Review Board, and all participants gave written informed consent prior to participation.

In this analysis, we included cognitively normal older adults who underwent their baseline 3 T MRI between 2009 and 2015. Five participants were excluded due to cognitive impairment at baseline, one participant due to Parkinson’s disease diagnosed during follow-up, and two participants due to pacemaker implementation. A total of 64 older participants were included at baseline, of which 39 participants returned for their second MR imaging visit, and 8 participants returned for a third MR imaging visit. The mean follow-up duration for imaging was 2.68 ± 0.87 years. We conducted an analysis to only include individuals with two time points due to the reduced number of individuals with second and third visit follow-ups. This was to understand whether this effect was robust to incomplete data.

### Neurocognitive assessments

The neuropsychological battery encompassed five domains [16, 17]: (1) Attention/Processing speed, (2) Executive function, (3) Language, (4) Memory, and (5) Visual-spatial ability (Additional file 1: Table S1). The scoring of the Trails Making Test-A and B tests were reversed such that higher scores in each test denoted better cognitive function. We standardized the score of every test in each time point by using the mean and standard deviation of the raw scores obtained from the baseline assessment. Domain-specific *z*-scores were calculated by averaging the standard scores across tests.

### MR image acquisition

MRI data was collected on a 3 T Siemens Trio scanner using a 12-channel head coil located at the MR Research Center at the University of Pittsburgh. High-resolution structural T1-weighted magnetization-prepared rapid gradient echo (MPRAGE) sequences were collected with TR = 2300 ms, TI = 900 ms, flip angle = 9°, FOV = 256 × 224 mm, 176 slices, and 1-mm isotropic voxels. Resting-state T2*-weighted blood oxygen-level-dependent (BOLD) signal was acquired by gradient-echo echo-planar imaging with TR = 2000 ms, TE = 34 ms, GOV = 128 × 128, 28 slices, and 2 × 2 × 4 mm voxel size. Participants were instructed to keep their eyes open and to fixate on a crosshair presented in the middle of the screen. T2-weighted fluid-attenuated inversion recovery (FLAIR) was acquired with TR = 9160 ms, TE = 90 ms [effective], TI = 2500 ms, FOV = 212 × 256, 48 slices, and 1 × 1 × 3 mm resolution with no slice gap.

### PiB-PET acquisition and analysis

PiB-PET acquisition and analyses followed a previously described and validated approach [18]. PiB was injected intravenously (12–15 mCi, over 20 s, specific activity ∼ 1–2 Ci/μmol), and PET image acquisition was performed at 50–70 min post-injection. MR images were utilized for co-registration and region of interest definitions. Standardized uptake value ratios (SUVR) were calculated as the ratio of regional PiB retention to that in the cerebellar gray matter. Regional cut-offs were determined with sparse k-means clustering in the anterior cingulate (cutoff 1.69), anterior ventral striatum (cutoff 1.60), and frontal (cutoff 1.65), lateral temporal (cutoff 1.56), parietal (cutoff 1.53), and precuneus cortices (cutoff 1.61) [19]. Individuals with SUVR values exceeding the cutoff point in any of these six regions was classified as PiB(+). Global SUVR values were calculated by weighted averaging of the six regional SUVR values.

FDG-PET acquisition and estimations of brain glucose metabolism have been described in previous work [20]. We used a similar approach to analyze FDG-PET data as we did for PiB. Summed FDG SUVR (relative to cerebellar gray matter) values were determined at 40–60 min post-injection and were corrected for cortical atrophy. We extracted the average FDG SUVR in the anterior cingulate, anterior ventral striatum, and frontal, lateral temporal, parietal, and precuneus cortices (same six regions extracted for PiB).

### APOE status

APOE genotype was derived from genotyping of isolated DNA from blood [9].

### Structural image preprocessing

Structural images were coregistered to the MPRAGE and segmented with SPM12’s multi-spectral segmentation, which generates a deformation field that can be used to normalize images to a standard anatomical space (MNI). This segmentation generates probability maps for gray matter, white matter, cerebrospinal fluid, skull, soft tissue, and air. We threshold gray, white, and CSF maps with a threshold of 0.1 to generate an automated mask to include only intracranial tissue.

We segmented the hippocampus using a FSL FIRST toolbox [21]—we extracted the total hippocampal volume with the MPRAGE. White matter hyperintensity (WMH) burden was quantified with a semi-automatic, fuzzy connectedness algorithm that segmented T2-weighted FLAIR images [22]. Both hippocampal volume and white matter hyperintensity burden were normalized by intracranial volume. Due to issues of normality, we used the log transformed value of WMH.

### Resting-state preprocessing

Resting-state fMRI preprocessing was conducted with the Statistical Parametric Mapping software (SPM12; http://www.fil.ion.ucl.ac.uk/spm/software/spm12/). Images underwent slice-time correction, motion correction, co-registration to the skull-stripped structural image, normalization with the generated deformation field, and smoothing with an 8-mm Gaussian kernel. To account for effects of no interest, we regressed the following features per voxel: 6 parameters of motion, 5 eigenvariates of white matter and cerebrospinal fluid (i.e., CompCor) [23], and sinusoids corresponding to unwanted frequencies outside of the band-pass in resting state (i.e., a band-pass filter 0.008–0.15 Hz). By doing this in one step, we do not reintroduce artifact/noise into our signal [24].

### Resting-state network connectivity

Brain networks were computed using template-based rotation (TBR) [25]. Seven brain network templates were selected from a normative sample [25]: default mode network/anterior salience network, cognitive control network, language network, left and right executive control networks, reward network, and memory encoding network. We used TBR to generate each network connectivity map per participant. TBR uses a set of pre-established template networks to constrain the parcellation of variance and extracts time courses that are highly correlated with the spatial pattern in the template—i.e., it extracts canonical time courses per network and generates a single voxel-wise connectivity map per network.

### Statistical analysis

To identify significant changes in connectivity longitudinally, we conducted seven voxel-wise paired *t*-tests in participants with two MRI scans (*N* = 39). This identified connectivity that significantly changed across time. We used statistical non-parametric mapping (SnPM13; http://warwick.ac.uk/snpm) [26] with permutation testing (10,000 permutations). To adjust for multiple comparisons, we controlled the cluster-wise (uncorrected cluster forming threshold at *p* < 0.001) family-wise error (FWE) rate at 0.05. We extracted connectivity in significant clusters for all participants at all time points and used that in subsequent analyses.

In the entire sample with every time point, we evaluated longitudinal associations with generalized estimating equations (GEEs). GEE is a type of regression analysis that includes cross-sectional (between-subjects) and longitudinal (within-subjects) relationships simultaneously. Furthermore, GEE can handle missing values and unequal follow-up times [27]. We assumed an exchangeable correlation matrix to account for repeated measurements. GEE was conducted by using Statistical Package for the Social Sciences version 19.0 (SPSS19.0) with the significance level set at 0.05. We did not utilize an AR-correlation matrix as it assumes that the interval between visits are the same and do not have the sample size to utilize an unstructured correlation matrix.

We investigated the association between connectivity and the following predictors: time, FDG SUVR (glucose metabolism), total hippocampal volume, normalized WMH volume, and PiB SUVR (amyloid)—each predictor’s interaction with time was modeled *only* if it was significant to avoid over-fitting. For each predictor, we used the data at each time point as they were measured longitudinally. We adjusted for sex, education, race, age, and total intracranial volume.

Similar to the connectivity, we investigated whether there were significant changes in cognitive function at baseline and over time. We modeled each cognitive domain (independently) and tested for a significant effect of time alone and then subsequently adjusted for sex, education, race, age, and total intracranial volume. We then investigated associations between each cognitive domain and connectivity, its interaction with amyloid and time—only significant interactions were kept in the model. The robustness of the three GEE models conducted above was provided in Additional file 1: Table S6.

Due to the low number of follow-ups at time 2, we conducted an analysis with only the *N* = 39 participants and two time points to test if these effects were robust to unequal follow-ups and missing data.

## Results

Sixty-four cognitively normal older participants were included at baseline for our analyses. The average age was 75.5 ± 6.2 years, and 28 (44.4%) participants were classified as PiB positive at baseline. Table 1 shows the complete baseline (and follow-up) demographic data.

**Table 1:** Demographic data and group comparison in participants at three time points

	Baseline	Visit 1	Visit 2
Variable	(*N* = 59)	(*N* = 40)	(*N* = 7)
Age at enrollment, mean (SD)	75.9 (6.2)	75.1 (6.4)	77.8 (5.5)
Male, no. (%)	19 (32.2%)	15 (37.5%)	2 (28.6%)
Years of education, mean (SD)	14.6 (2.4)	14.7 (2.6)	14.6(2.8)
Mini-Mental State Examination, mean (SD)	28.7 (1.4)	28.7 (1.5)	28.4 (1.5)
Race/ethnicity, no. (%)			
White	49 (83.1%)	32 (80.0%)	5 (71.4%)
Black	9 (15.3%)	7 (17.5%)	1 (14.3%)
Asian	1 (1.7%)	1 (2.5%)	1(14.3%)
APOE ε4 allele carrier, no. (%)			
Carrier	11 (18.6%)	28 (70.0%)	1(14.3%)
Non-carrier	38 (64.4%)	10 (25.0%)	6 (85.7%)
Data unavailable	10 (16.9%)	2 (14.3%)	0
Global PiB SUVR, mean (SD)	1.5 (0.4)	1.5 (0.3)	1.6 (0.6)
PiB status, no. (%)			
Baseline			
PiB(+)	25 (42.4%)	15 (37.5%)	3 (42.9%)
PiB(−)	34 (57.6%)	25 (62.5%)	4 (57.1%)
Intracranial volume (cubic mm)	1,771,813 (187,320.3)	1,814,635 (209,336.6)	1,888,776 (248,791)
Glucose metabolism (FDG SUVR)	1.1(0.1)	1(0.1)	1.1(0.1)
Normalized hippocampal volume	0.01 (0.04)	0.01 (0.00)	0.03 (0.00)
Normalized WMH volume	2.5 (0.6)	2.4(0.6)	2.5(0.5)

**Fig. 1:**
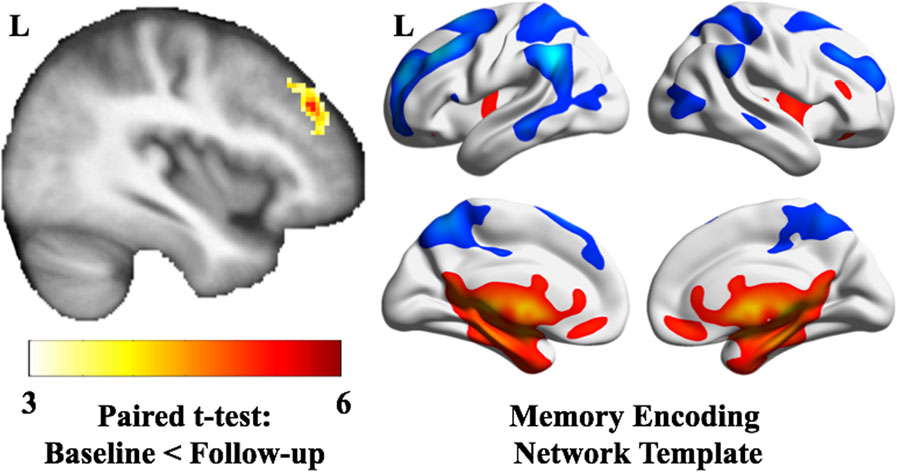
(Left) Resting-state connectivity of the left middle frontal gyrus increased from baseline to follow-up in the memory encoding network—this is the template used to extract the network. Colors indicate *t*-test values (only significant values are shown). Data is overlaid on an average structural image from this sample. (Right) The memory encoding network template was used in combination with template-based rotation to extract connectivity of the memory encoding network per participant. Colors indicate areas of greater connectivity, where red areas indicate regions of typically strong connectivity with the network and blue areas indicate regions of typically weak connectivity with the network

We investigated seven networks of interest and found that only the left middle frontal gyrus (MFG) connectivity within the memory encoding network (MEN) significantly increased over time (Fig. 1). No other networks significantly increased or decreased in connectivity over time. We extracted the connectivity of the left MFG and modeled it using GEE in the full sample (Table 2).

**Table 2:** Generalized estimation equation results modeling significant changes over time (mean duration of 2 years) in connectivity. MFG connectivity significantly increased by 0.264 units per year, which was lower by 0.1 units per unit PiB SUVR. *p < 0.05; **p < 0.01; ***p < 0.001

Predicting changes in MFG connectivity with memory encoding network		
	*β* (unstandardized)	Standard error
Intercept	− 1.532	1.976
Covariates of no interest		
Sex (M:F)	− 0.012	0.123
Education (years)	0.015	0.018
Race (B:W)	0.032	0.253
Age (years)	0.015	0.010
Intracranial volume (cubic mm)	0.000	0.000
Predictors		
Time (years)	0.264*	*0.065*
amyloid (PiB SUVR)	− 0.049	0.237
glucose metabolism (FDG SUVR)	− 0.034	0.516
Normalized hippocampal volume	− 13.853	61.567
Normalized WMH volume	0.063	0.092
Interactions		
Time × PiB	− 0.100*	*0.031*
Time × FDG	Not significant, not included	
Time × hippocampal volume	Not significant, not included	
Time × WMH volume	Not significant, not included	

**Fig. 2:**
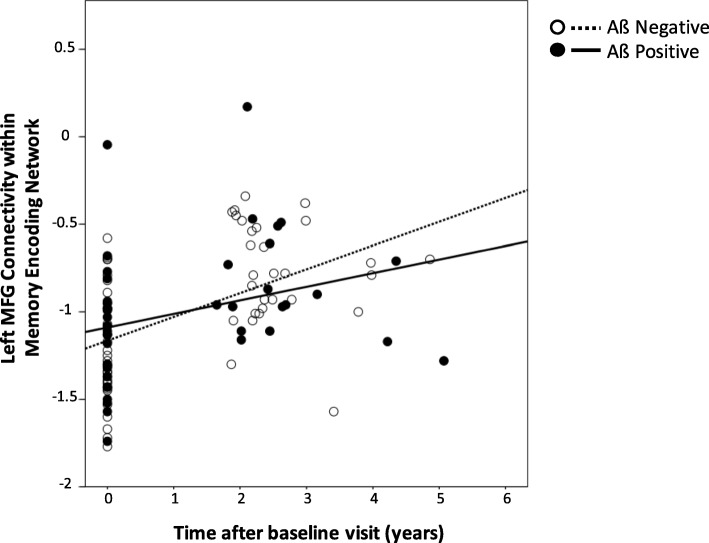
Left middle frontal gyrus connectivity in the memory encoding network increased significantly by 0.264 units per year—each unit of PiB SUVR decreased this rate by 0.1. While our analyses used continuous amyloid as measured by PiB, we used the definitions of Aß negative and positive as groupings. Time after baseline visit is measured continuously

We first investigated what factors were associated with these changes in connectivity. Left MFG connectivity increased every year, but this rate of change was slower in those with greater PiB (Table 2, Fig. 2). None of the following variables were associated with change in connectivity: sex, education, race, age, intracranial volume, glucose metabolism, normalized hippocampal volume, and normalized WMH volume. This model explained 27% of the variance in connectivity (see Additional file 1: Figure S1 for diagnostic plots).

As an exploratory analysis, we investigated whether the association with global PiB was specific to particular regional PiB: we replaced the global PiB SUVR with six regional PiB SUVR and conducted the same GEE analysis in an exploratory way. We found connectivity increased every year, but this rate of change was slower in individuals with greater PiB in anterior cingulate gyrus, anteroventral striatum, and precuneus (Additional file 1: Table S5). Since APOE4 presence is associated with high amyloid deposition [28], we replaced amyloid in our model with APOE4 status and found that APOE4 was not significantly associated with connectivity change. When we tested the robustness of this effect to sample size and missing data (*N* = 39 with two time points), we found similar changes over time in resting-state connectivity dependent on PiB (Additional file 1: Table S2); however, the interaction between PiB and longitudinal changes in connectivity was no longer significant but the effect size differed only slightly (*β* = − 0.1 to *β* = − 0.083).

We then investigated if changes in cognitive function existed in our cohort. When modeling the effect of time on cognitive function (without adjustment), we found that there was a significant decrease in visuospatial function over time but not in other cognitive domains (Additional file 1: Table S3). These were not significant after adjusting for sex, education, race, age, and total intracranial volume (Additional file 1: Table S4). Greater age was associated with lower cognitive function in language, visuospatial, attention, and executive function domains. When we tested these findings’ robustness to sample size and follow-up, we found no differences in effect sizes or significance.

**Fig. 3:**
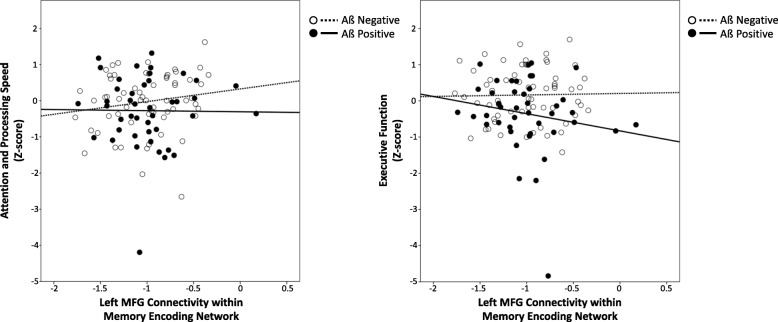
An interactive effect of amyloid deposition (PiB) was found in the relationship between left middle frontal gyrus connectivity in the memory encoding network and cognitive function (attention/processing speed, left; executive function, right). While our analyses used continuous amyloid as measured by PiB, we used the definitions of Aß negative and positive as groupings

We then modeled the association between cognitive function and connectivity. The final model for attention and processing speed explained 36% of its variance and the final model for executive function explained 37% of its variance (see Additional file 1: Figure S1 for diagnostic plots). Functional connectivity was not directly associated with baseline cognitive function or with changes in cognitive function over time. In those with lower amyloid, greater connectivity was associated with better attention/processing speed and executive function. However, in those with greater amyloid, greater connectivity was associated with worse attention/processing speed and executive function with greater levels of amyloid (Table 3, Fig. 3). This connectivity by amyloid interaction effect was not time-dependent (i.e., the association held at each time point but did not vary by time) and was significant after adjusting for demographic data and all other neurodegeneration markers (FDG SUVR, total hippocampal volume, and normalized WMH volume). When we tested these findings robustness to sample size and follow-up, we found no differences in effect sizes or significance.

**Table 3:** Generalized estimation equation results show the interactive effect of left MFC connectivity and amyloid deposition on two specific cognitive domains, attention/processing speed, and executive function, adjusting for demographic data, neurodegenerative biomarkers, and time effect. *p < 0.05; **p < 0.01; ***p < 0.001

	*β* (unstandardized)	Standard error
Predicting changes in attention and processing speed cognitive domain		
Intercept	0.128	3.304
Sex (M:F)	− 0.511*	*0.241*
Education (years)	0.077	0.043
Race (B:W)	0.884*	*0.413*
Age (years)	− 0.051	0.024
Intracranial volume (cubic mm)	0.000*	*0.000*
Time (years)	− 0.011	0.047
glucose metabolism (FDG SUVR)	− 0.054	0.899
Normalized hippocampal volume	309.237*	*129.011*
Normalized WMH volume	0.471	0.345
amyloid (PiB SUVR)	− 0.844*	*0.382*
MFG connectivity with memory encoding network	1.815**	*0.524*
Amyloid × MFG connectivity	− 1.056***	*0.292*
Predicting changes in executive function cognitive domain		
Intercept	5.544*	*2.261*
Sex (M:F)	− 0.357	0.243
Education (years)	0.055	0.041
Race (B:W)	− 0.292	0.424
Age (years)	− 0.055*	*0.022*
Intracranial volume (cubic mm)	0.000	0.000
Time (years)	0.022	0.060
glucose metabolism (FDG SUVR)	0.716	0.833
Normalized hippocampal volume	186.491	131.311
Normalized WMH volume	0.595*	*0.247*
amyloid (PiB SUVR)	− 2.205***	*0.611*
MFG connectivity with memory encoding network	1.926***	*0.511*
Amyloid × MFG connectivity	− 1.384***	*0.330*

In the model predicting changes in attention and processing speed, significant variables include sex (p=0.034), age (p=0.031), race (p=0.032), ICV (p=0.012), normalized hippocampal volume (p=0.012), amyloid (p=0.027), MFG connectivity with memory encoding network(p=0.001), and amyloid x MFG connectivity (p< 0.001) ; In the model predicting changes in executive function, significant variables include age (p=0.013), amyloid (p< 0.001), MFG connectivity with memory encoding network (p< 0.001) and amyloid × MFG connectivity (p< 0.001).

## Discussion

In cognitively intact older adults, we demonstrated that resting-state functional connectivity increased significantly in the left middle frontal gyrus (MFG) within the memory encoding network (MEN), and the rate of change in connectivity was lower in participants with greater amyloid deposition. In those with lower amyloid, greater functional connectivity in left MFG was associated with better attention/processing speed and executive function. However, this association was reversed in those with greater amyloid deposition. We found that cognitive function in this cohort was overall relatively stable over 2 years with only slight decline in one domain. This evidence together suggests that greater functional connectivity may help offset the effects of amyloid on cognitive function in this early stage (since cognitive function was positively associated with connectivity in those with lower amyloid). This process maintains healthy cognitive function as long as possible, but these mechanisms may begin to fail in the later stages as pathology accumulates (since cognitive function was negatively associated with connectivity in those with greater amyloid).

Our findings support previous models of increased activation and functional connectivity associated with healthy aging and preclinical stages of AD. This increased functional connectivity has previously been described using a compensatory model, such that higher levels of activity or connectivity are thought to preserve prior levels of cognitive function [29]. This compensatory function of increased activation or connectivity likely depends on the context. For example, when the system is at prime functioning dynamic (i.e., young adulthood), lower activation is associated with better performance (neural efficiency model) [30]. It is important to interpret this result cautiously as when we conducted an analysis to test this effect robustness (by including only the 39 participants with two time points), this effect was no longer significant though the effect size was only slightly reduced. This is a clear limitation of this result, and future studies are needed to understand this effect.

We also found that greater MFG connectivity was associated with better attention/processing speed and executive function in individuals with lower amyloid but was associated with worse cognitive function in those with greater amyloid. The MFG is of particular interest since it seems to accumulate amyloid at a very early stage, which is associated with hypoconnectivity with the default mode and frontoparietal network [31]. These results may explain some discrepancy in the literature regarding associations between connectivity and cognitive function. In a study with cognitively normal older adults who were all PiB negative, greater amyloid deposition was associated with greater dynamic functional connectivity primarily in the default mode network, which in turn was associated with better overall cognitive function (measured by MMSE) [32]. However, these authors also found that in a population with even lower levels of amyloid accumulation (primarily those who were PiB negative and normal Aβ42 peptide in cerebrospinal fluid) that greater amyloid was instead associated with greater connectivity within the same network. We have also previously shown in this cohort that in those who were PiB positive, lower episodic memory was associated with greater default mode connectivity [9]. Our current study may help explain this discrepancy that it may be dependent on the pathological state of amyloid in the brain.

We found that connectivity increases over time but may increase at a slower rate in those who have a more severe amyloid pathology. Connectivity may be compensatory in the early stages but could possibly lead to more rapid accumulation of amyloid in the long-term—a vicious downward spiral. Previous studies have shown that areas of high amyloid accumulation are cortical hubs—or regions of high connectivity at rest that may act as information integration centers [33]. For example, neurostimulation in mouse models increases extracellular Aβ [34] and synaptic transmission increases amyloid precursor protein endocytosis [35] and aerobic glycolysis [36], precipitating the aggregation of amyloid in the brain. This may explain past findings that show that network hyperconnectivity may herald subsequent amyloid-related cognitive decline [37]. Our results help integrate these findings by showing a greater longitudinal increase in functional connectivity in those with low amyloid burden. Connectivity may be compensatory with regard to amyloid but may become less effective for maintaining cognitive function in the long term.

We found that connectivity was primarily associated with attention/processing speed and executive function. This is in accordance with prior studies which have shown that processing speed, executive function, and episodic memory are impaired in the earliest preclinical stages [38]. This may suggest that pathology impacts these functions early on and hence a need for neural compensation in connectivity within these domains.

We hypothesize that the level of activation or connectivity serves to maintain the dynamic equilibrium or homeostasis of the functioning network—though we do not test this explicitly. The presence of amyloid in the present results is associated with a diminishment of expected increase in connectivity, which may represent a decreased homeostatic drive. One would anticipate that this would be associated with a decrease in cognitive function in those with greater amyloid. However, we suggest that the increase in connectivity, albeit diminished, is still sufficient to maintain cognitive functioning for the time being. The association between cognitive function and connectivity especially in those with lower amyloid (independent of time) may further demonstrate this effect. Longer follow-up and larger samples may be necessary to observe the cognitive and clinical effects of this decreased neural homeostasis.

In an exploratory analysis, we investigated whether the association between connectivity and global PiB was specific to particular regions. We conducted an analysis that included regional PiB instead of global PiB and found that while connectivity increased over time it was lower in individuals with greater amyloid in the anterior cingulate gyrus, anteroventral striatum, and precuneus. The anterior cingulate and precuneus are nodes of the default mode network, which has been previously implicated in Alzheimer’s disease pathology. Striatal amyloid pathology however is more commonly attributed to early onset Alzheimer’s or Down’s syndrome. These three regions may be hubs in which deposition of amyloid renders the most functional disturbance in the aging brain. However, this result should be interpreted with extreme caution since it is a highly exploratory analysis.

Prevention and treatment of AD remains a major public health challenge. Since the development of amyloid imaging, it has been possible to track preclinical AD and thus identify factors that may accelerate or delay the progression from the presence of cerebral amyloid positivity to clinical AD. A primary focus of many interventions has been the removal of cerebral amyloid, with the hope that this would disrupt the neurodegenerative cascade. Unfortunately, amyloid-targeted treatment studies have not yet shown a clear clinical benefit. It is becoming more imperative to identify other mechanisms for delaying or dampening the progressive neural degeneration. Studies that identify neural system correlates of AD risk may provide clues to these mechanisms and serve as biomarkers for testing interventions. For instance, we hypothesize that interventions that can be shown to enhance functional connectivity may restore neural system homeostasis. We suspect the benefits of interventions that are known to delay AD progression can be tracked through their effects on functional connectivity.

There are several notable limitations in our study. In our current sample, we did not observe overt cognitive decline and clinical effects of decreased neural system homeostasis over this short follow-up time. It is important to include individuals with and without mild cognitive impairment, i.e., including individuals in a preclinical stage who transition to mild cognitive impairment. Furthermore, we are limited by attrition over time. After an analysis to test robustness, the effect of amyloid by time was no longer significant although the effect size was similar indicating that this may be due to inability to detect small effect sizes. This is a major limitation of that finding and should be interpreted cautiously. Clearly, future studies need to properly power and test this hypothesis. We were limited by the lack of observed onset in amyloid deposition; thus, it is unclear how long an individual has been maintaining cognitive performance in the context of amyloid toxicity. We anticipate that having a longer follow-up and larger sample size may provide more insight for these two limitations in the future. We did not measure tau deposition in this cohort. In cognitively normal elderly participants with amyloid deposition, network hyperconnectivity and hypoconnectivity was dependent on tau deposition [39]. Future studies should investigate these associations and their interactions. While we did not explicitly enrich our dataset for PiB-positive individuals, our recruitment strategies resulted in a higher proportion of PiB-positive individuals than the general population, which may affect the results of our study. We did not model any quadratic terms due to the limited sample size and follow-up, but future studies should investigate whether these effects are better modeled with higher-order effects (e.g., quadratic time).

## Conclusion

In conclusion, we demonstrated that the resting-state functional connectivity of older participants increased significantly over 2 years in the left middle frontal gyrus (MFG) within the memory encoding network (MEN). Participants with greater amyloid deposition experienced a diminished increase in functional connectivity compared to those with lower amyloid deposition. Our results suggest that early alterations of network connectivity may be detected before overt cognitive decline. These alterations may serve as a mechanism for maintaining homeostasis in the context of age-related changes, amyloid deposition, or other neurodegenerative changes. It may be important to understand when this change occurs, future studies should investigate the suprathreshold stage of pathology where connectivity fails to compensate for cognitive function.

## Additional file

Additional file 1: **Table S1.** The cognitive domains and the constituent neuropsychological test battery. **Table S2.** Generalized estimation equation results modeling significant changes over time (mean duration of 2 years) in connectivity in 39 participants with more than two visits in the cohort. **Table S3.** Generalized estimation equation results modeling changes in cognitive domains and time (without adjustment for other factors). **Table S4.** Generalized estimation equation results modeling changes in cognitive domains. **Table S5.** Generalized estimation equation results modeling the association between MFG connectivity and time as well as the regional effects of PiB SUVR adjusting for demographic and other neurodegenerative markers. **Figure S1.** The scatter plots for the explained variance and homoscedastic check of the three GEE models in our analysis (Connectivity, attention/processing speed, and executive function). (Top Left) Predicted connectivity vs. actual connectivity; (Top Right) Standardized predicted connectivity vs. standardized residual; (Middle Left) Predicted attention and processing speed vs. actual attention and processing speed; (Middle Right) Standardized predicted attention and processing speed vs. standardized residual; (Bottom Left) Predicted executive function vs. actual executive function; (Middle Right) Standardized predicted executive function vs. standardized residual.

## Data Availability

All data is available by request.

## Abbreviations

AD: Alzheimer’s disease
APOE4: Apolipoprotein E gene who may have greater frequency for AD
Aβ: Amyloid-beta (peptides that are the main component of the amyloid plaques)
BOLD: Blood oxygen-level dependent
DMN: Default mode network
FDG: Fluorodeoxyglucose (a PET tracer for measuring glucose metabolism)
FLAIR: Fluid-attenuated inversion recovery (a structural MRI sequence used to detect hyperintensities in the white matter)
MCI: Mild cognitive impairment
MEN: Memory encoding network (see Fig. 1) identified using resting-state independent component analysis with hippocampus, thalamus, parahippocampus, amygdala, and prefrontal cortex as primary nodes of the network
MFG: Middle frontal gyrus
MMSE: Mini-Mental State Examination
MPRAGE: Magnetization-prepared rapid gradient echo (a structural MRI sequence used primarily to identify gray/white matter)
MRI: Magnetic resonance imaging
PET: Positron emission tomography
PiB: Pittsburgh compound B (a PET tracer for measuring amyloid)
PiB(−): PiB negative: a designation indicating a low level of amyloid as measured by PiB-PET
PiB(+): PiB positive: a designation indicating a significant presence of amyloid as measured by PiB-PET
SUVR: Standardized uptake value ratios
WMH: White matter hyperintensities
