# Supplementary material for: The effect of amyloid deposition on longitudinal resting-state functional connectivity in cognitively normal older adults
Source: Alzheimers Res Ther. 2020 Jan 6;12:7. doi: 10.1186/s13195-019-0573-1 (PMC6945413; doi:10.1186/s13195-019-0573-1)
Supplement: Supplementary file 1 — Table S1. The cognitive domains and the constituent neuropsychological test battery. Table S2. Generalized estimation equation results modeling significant changes over time (mean duration of 2 years) in connectivity in 39 participants with more than two visits in the cohort. Table S3. Generalized estimation equation results modeling changes in cognitive domains and time (without adjustment for other factors). Table S4. Generalized estimation equation results modeling changes in cognitive domains. Table S5. Generalized estimation equation results modeling the association between MFG connectivity and time as well as the regional effects of PiB SUVR adjusting for demographic and other neurodegenerative markers. Figure S1. The scatter plots for the explained variance and homoscedastic check of the three GEE models in our analysis (Connectivity, attention/processing speed, and executive function). (Top Left) Predicted connectivity vs. actual connectivity; (Top Right) Standardized predicted connectivity vs. standardized residual; (Middle Left) Predicted attention and processing speed vs. actual attention and processing speed; (Middle Right) Standardized predicted attention and processing speed vs. standardized residual; (Bottom Left) Predicted executive function vs. actual executive function; (Middle Right) Standardized predicted executive function vs. standardized residual. [file 13195_2019_573_MOESM1_ESM.docx]

**The Effect of Amyloid Deposition on Longitudinal Resting-State Functional Connectivity in Cognitively Normal Older Adults**

Chemin Lin^1,2 *^, Maria Ly^2*^, Helmet T. Karim^2^, Wenjing Wei^4,5^, Beth E. Snitz^3^, William E. Klunk^2, 3^, Howard J. Aizenstein^2,6^

1. Department of Psychiatry, Keelung Chang Chung Memorial Hospital, Keelung, Taiwan
2. Department of Psychiatry, University of Pittsburgh, Pittsburgh PA, USA
3. Department of Neurology, University of Pittsburgh, Pittsburgh PA, USA
4. The Third Xiangya Hospital of Central South University, Changsha, Hunan, China
5. Shanghai Mental Health Center, Shanghai Jiao Tong University School of Medicine, Shanghai, China
6. Department of Bioengineering, University of Pittsburgh, Pittsburgh PA, USA

*Both authors serve as co-primary authors for this manuscript.

Correspondence to:

Howard J. Aizenstein, MD, PhD

Western Psychiatric Institute and Clinic

3811 O’Hara Street

Pittsburgh, PA 15213

P: 412-246-6048

F: 412-246-6030

E: [haizenstein@upmc.edu](mailto:haizenstein@upmc.edu)

For submission to Alzheimer’s Research & Therapy

Abstract word count = 233/350

Total word count = 3355/NA

Tables and Figures = 6/NA

References = 39/NA

**Acknowledgements:** This study was supported by funding from NIA P50 AG005133, RF1 AG025516, P01 AG025204, 5K23AG038479, R01 MH076079, NIA T32 AG021885. and NIMH T32 MH019986.

**Conflicts of Interest:** GE Healthcare holds a license agreement with the University of Pittsburgh based on the technology described in this manuscript. Dr. Klunk is a co-inventor of PiB and, as such, has a financial interest in this license agreement. GE Healthcare provided no grant support for this study and had no role in the design or interpretation of results or preparation of the manuscript. The other authors declare no conflicts of interest.

Key words: preclinical Alzheimer’s disease, resting state fMRI, longitudinal, compensation, homeostatic regulation, amyloid, PiB, connectivity

**Description:** Supplemental tables describing the cognitive battery (supplemental table 1); sensitivity analysis focusing on the 39 participants with just two time points (supplemental table 2); associations between changes in cognitive function and time as well as other demographic features (supplemental table 3 and 4); GEE analysis replacing global PiB with regional values of PiB (only shows estimates of regional PiB and its interaction with time) (supplemental table 5); and diagnostic plots of the three GEE models in the main manuscript (supplemental figure 1).

| Table S1. The cognitive domains and the constituent neuropsychological test battery | |
| --- | --- |
| Domain | Neuropsychological task |
| Attention/Processing speed | Trail making test part A |
|  | Digit span forward |
| Executive function | Trail making test part B |
|  | Digit symbol substitution task |
|  | paper and the pencil version of the Stroop Color Word test |
| Language | Category fluency (Animals) |
|  | Letter fluency (FAS) |
|  | Boston Naming Test (60 item) |
| Memory | Consortium to Establish a Registry for AD Word List Recall trial 1 |
|  | Modified Rey Osterrieth figure delayed recall |
|  | Logical Memory Story A from the Wechsler Memory Scale – Revised |
| Visual-spatial | Modified block design (total score) |
|  | Modified Rey Osterrieth Figure copy |

Table S2. Generalized estimation equation results modeling significant changes over time (mean duration of 2 years) in connectivity in 39 participants with more than two visits in the cohort. The estimate of the change over time is still significant with similar effect size, the effect of amyloid however is no longer significant (unstandardized estimate -0.083) suggesting that this may be due to lack of power to detect this effect.

| **Predicting Changes in MFG Connectivity with Memory Encoding Network** | | |
| --- | --- | --- |
| **Covariates of no interest** | **β (unstandardized)** | **p-value** |
| Sex (M: F) | -0.062 | >0.05 |
| Education (years) | 0.017 | >0.05 |
| Race (B: W) | 0.099 | >0.05 |
| Age at Baseline (years) | 0.014 | >0.05 |
| Intracranial Volume (cubic mm) | 0.000 | >0.05 |
| **Predictors** |  |  |
| **Time (years) *** | **0.264** | **<0.05** |
| Amyloid (PiB SUVR) | 0.100 | >0.05 |
| Glucose Metabolism (FDG SUVR) | 0.040 | >0.05 |
| Normalized Hippocampal Volume | 23.215 | >0.05 |
| Normalized WMH Volume | 0.096 | >0.05 |
| **Interactions** |  |  |
| **Time x PiB *** | -0.083 | >0.05 |
| Time x FDG | Not Significant, Not Included | |
| Time x Hippocampal Volume | Not Significant, Not Included | |
| Time x WMH Volume | Not Significant, Not Included | |

Table S3. Generalized estimation equation results modeling changes in cognitive domains and time (without adjustment for other factors).

|  | **Predicting Changes in**  **Language Cognitive Domain** | |
| --- | --- | --- |
|  | **β (unstandardized)** | **p-value** |
| Time (years) | -0.035 | >0.05 |
|  |  |  |
|  | **Predicting Changes in**  **Memory Cognitive Domain** | |
|  | **β (unstandardized)** | **p-value** |
| Time (years) | 0.011 | >0.05 |
|  |  |  |
|  | **Predicting Changes in**  **Visual-spatial Cognitive Domain** | |
|  | **β (unstandardized)** | **p-value** |
| Time (years) | -0.091 | **<0.05** |
|  |  |  |
|  | **Predicting Changes in**  **Attention Cognitive Domain** | |
|  | **β (unstandardized)** | **p-value** |
| Time (years) | -0.039 | >0.05 |
|  |  |  |
|  | **Predicting Changes in**  **Executive Function Cognitive Domain** | |
|  | **β (unstandardized)** | **p-value** |
| Time (years) | -0.049 | >0.05 |

Table S4. Generalized estimation equation results modeling changes in cognitive domains. While several were associated with age at baseline, none of these domains significantly changed across time.

|  | **Predicting Changes in**  **Language Cognitive Domain** | |
| --- | --- | --- |
|  | **β (unstandardized)** | **p-value** |
| Sex (M:F) | 0.298 | >0.05 |
| Education (years) | -0.003 | >0.05 |
| Race (B:W) | 0.126 | >0.05 |
| **Age at Baseline (years) *** | **-0.046** | **<0.05** |
| Intracranial Volume (cubic mm) | 0.000 | >0.05 |
| Time (years) | 0.007 | >0.05 |
|  |  |  |
|  | **Predicting Changes in**  **Memory Cognitive Domain** | |
|  | **β (unstandardized)** | **p-value** |
| Sex (M:F) | -0.297 | >0.05 |
| Education (years) | 0.064 | >0.05 |
| Race (B:W) | -0.158 | >0.05 |
| Age at Baseline (years) | -0.002 | >0.05 |
| Intracranial Volume (cubic mm) | 0.000 | >0.05 |
| Time (years) | -0.015 | >0.05 |
|  |  |  |
|  | **Predicting Changes in**  **Visual-spatial Cognitive Domain** | |
|  | **β (unstandardized)** | **p-value** |
| Sex (M:F) | 0.224 | >0.05 |
| Education (years) | 0.061 | >0.05 |
| **Race (B:W) *** | **-0.789** | **<0.05** |
| **Age at Baseline (years) *** | **-0.042** | **<0.05** |
| Intracranial Volume (cubic mm) | 0.000 | >0.05 |
| Time (years) | -0.059 | >0.05 |
|  |  |  |
|  | **Predicting Changes in**  **Attention Cognitive Domain** | |
|  | **β (unstandardized)** | **p-value** |
| Sex (M:F) | -0.374 | >0.05 |
| Education (years) | 0.049 | >0.05 |
| **Race (B:W) *** | **0.573** | **<0.05** |
| **Age at Baseline (years) *** | **-0.045** | **<0.05** |
| Intracranial Volume (cubic mm) | 0.000 | >0.05 |
| Time (years) | -0.001 | >0.05 |
|  |  |  |
|  | **Predicting Changes in**  **Executive Function Cognitive Domain** | |
|  | **β (unstandardized)** | **p-value** |
| Sex (M:F) | -0.376 | >0.05 |
| Education (years) | 0.047 | >0.05 |
| Race (B:W) | 0.428 | >0.05 |
| **Age at Baseline (years) *** | **-0.053** | **<0.05** |
| Intracranial Volume (cubic mm) | 0.000 | >0.05 |
| Time (years) | 0.006 | >0.05 |

Table S5. Generalized estimation equation results modeling the association between MFG connectivity and time as well as the regional effects of PiB SUVR adjusting for demographic and other neurodegenerative markers.

| **Predicting Changes in MFG Connectivity with Memory Encoding Network** | | |
| --- | --- | --- |
|  | **β (unstandardized)** | **Standard Error** |
| **Regional amyloid PiB** |  |  |
| **Time x PiB (ACG)** | **-0.092**** | **0.034** |
| **Time x PiB (AVS)** | **-0.102**** | **0.037** |
| Time x PiB (FRC) | -0.046 | 0.188 |
| Time x PiB (LTC) | -0.100 | 0.031 |
| Time x PiB (PAR) | -0.056 | 0.032 |
| **Time x PiB (PRC)** | **-0.066*** | **0.029** |

anterior cingulate gyrus (ACG); anteroventral striatum (AVS), frontal cortex (FRC); lateral temporal cortex (LTC); parietal (PAR) and precuneus (PRC); * p < 0.05; ** p < 0.01; *** p < 0.001.

 Figure S1. The scatter plots for the explained variance and homoscedastic check of the three GEE models in our analysis (Connectivity, attention/processing speed, and executive function). (Top Left) Predicted connectivity vs. actual connectivity; (Top Right) Standardized predicted connectivity vs. standardized residual; (Middle Left) Predicted attention and processing speed vs. actual attention and processing speed; (Middle Right) Standardized predicted attention and processing speed vs. standardized residual; (Bottom Left) Predicted executive function vs. actual executive function; (Middle Right) Standardized predicted executive function vs. standardized residual
